# Mobile circular DNAs regulating memory and communication in CNS neurons

**DOI:** 10.3389/fnmol.2023.1304667

**Published:** 2023-12-06

**Authors:** Neil R. Smalheiser

**Affiliations:** Department of Psychiatry, University of Illinois College of Medicine, Chicago, IL, United States

**Keywords:** extrachromosomal circular DNA, double-strand breaks, DNA repair, immediate-early genes, transposable elements, secretory exosomes, transcription, neuronal plasticity

## Abstract

Stimuli that stimulate neurons elicit transcription of immediate-early genes, a process which requires local sites of chromosomal DNA to form double-strand breaks (DSBs) generated by topoisomerase IIb within a few minutes, followed by repair within a few hours. Wakefulness, exploring a novel environment, and contextual fear conditioning also elicit turn-on of synaptic genes requiring DSBs and repair. It has been reported (in non-neuronal cells) that extrachromosomal circular DNA can form at DSBs as the sites are repaired. I propose that activated neurons may generate extrachromosomal circular DNAs during repair at DSB sites, thus creating long-lasting “markers” of that activity pattern which contain sequences from their sites of origin and which regulate long-term gene expression. Although the population of extrachromosomal DNAs is diverse and overall associated with pathology, a subclass of small circular DNAs (“microDNAs,” ∼100–400 bases long), largely derives from unique genomic sequences and has attractive features to act as stable, mobile circular DNAs to regulate gene expression in a sequence-specific manner. Circular DNAs can be templates for the transcription of RNAs, particularly small inhibitory siRNAs, circular RNAs and other non-coding RNAs that interact with microRNAs. These may regulate translation and transcription of other genes involved in synaptic plasticity, learning and memory. Another possible fate for mobile DNAs is to be inserted stably into chromosomes after new DSB sites are generated in response to subsequent activation events. Thus, the insertions of mobile DNAs into activity-induced genes may tend to inactivate them and aid in homeostatic regulation to avoid over-excitation, as well as providing a “counter” for a neuron’s activation history. Moreover, activated neurons release secretory exosomes that can be transferred to recipient cells to regulate their gene expression. Mobile DNAs may be packaged into exosomes, released in an activity-dependent manner, and transferred to recipient cells, where they may be templates for regulatory RNAs and possibly incorporated into chromosomes. Finally, aging and neurodegenerative diseases (including Alzheimer’s disease) are also associated with an increase in DSBs in neurons. It will become important in the future to assess how pathology-associated DSBs may relate to activity-induced mobile DNAs, and whether the latter may potentially contribute to pathogenesis.

## Introduction and hypothesis

It has long been recognized that DNA has desirable properties to store and read out information, including its specific sequences, stability, and epigenetic modifications. Diverse molecules have been shown to have messenger actions within the CNS, ranging from amino acids and peptides to gases, nucleotides and even small RNAs, yet so far, genomic DNA has been exempt from this list because it is thought to function entirely within chromosomes in the nucleus. However, there are strong reasons to believe that mobile DNAs are formed and released from chromosomes during normal neuronal physiology:

Stimuli that activate the firing of neurons lead to the rapid transcription of immediate-early genes (e.g., Fos, Arc, FosB, Egr1, Npas4). Transcription of this subset of genes requires the formation of double-strand DNA breaks (DSBs) generated by topoisomerase IIb within the gene promoters within a few minutes, followed by repair within a few hours (Crowe et al., 2006; Madabhushi et al., 2015; Pollina et al., 2023). Transcription of heat shock genes, serum-induced immediate-early genes and nuclear receptor-activated genes in a variety of cell types also requires DNA double-strand breaks (Calderwood, 2016). A learning paradigm in living mice, contextual fear conditioning, also shows an increase in DSB formation which is a bit more widespread than seen in cultured neurons (including at sites of synaptic genes), and repair takes a bit longer (up to 24 h) (Li et al., 2019; Navabpour et al., 2020; Stott et al., 2021; Delint-Ramirez et al., 2022; Weber Boutros et al., 2022). DSB formation and repair appear to be involved in aspects of long-term retention of fear avoidance memory, insofar as agents which block DSB formation or repair inhibit aspects of the long-term memory (Navabpour et al., 2020). Other physiologic conditions which induce DSBs include exploration of a novel environment (Suberbielle et al., 2013) and wakefulness, with sleep being needed for repair of the DSBs to occur (Bellesi et al., 2016; Zada et al., 2019, 2021). DNA strand breaks also occur in brain somatic gene recombination (Kaeser and Chun, 2020).

The need to make double-stranded breaks in chromosomal DNA makes sense at a mechanistic level [relieving torsion that allows transcription of the affected gene to proceed (Calderwood, 2016)], but this process remains somewhat paradoxical and counterintuitive, since DSBs had been thought of as sites of potential genotoxic damage, and the repair mechanisms present within postmitotic (non-replicating) neurons are error-prone, so it is surprising for DSBs to not only occur normally but be required for expression of activity-dependent genes (Madabhushi et al., 2015).

A new insight into this process comes from a separate line of investigation carried out in non-neural systems, in which DSBs undergoing repair can be associated with the formation of extrachromosomal circular DNA (eccDNA) molecules [reviewed in Ling et al. (2021)]. This is exciting since, in the context of neuronal activation, formation of eccDNAs which contain sequences from their sites of origin would have potential physiological roles as long-lasting extrachromosomal “markers” and regulators of that activity pattern.

Extrachromosomal DNA molecules have been detected in brain and many other tissues (Shibata et al., 2012; Ling et al., 2021; Zhao et al., 2022; Guo et al., 2023; Zhong et al., 2023). They are enriched in specific areas (hotspots), including untranslated (3′-UTR and 5′-UTR) areas, regions with a high GC content, and transcriptionally active chromatin and can include both repeat elements and unique sequences. EccDNAs can arise from multiple mechanisms (Ling et al., 2021), assume both linear and circular forms, can be relatively large (> 1 megabase), can be derived from apoptotic or dying cells, and can contain entire genes that are amplified within cancer cells (Paulsen et al., 2018). Thus the population of extrachromosomal DNAs is quite diverse and overall associated with pathology. However, a subclass of small circular DNAs (“microDNAs,” ∼100–400 bases long), largely derives from unique genomic sequences and has attractive features to act as stable, mobile circular DNAs to regulate gene expression in a sequence-specific manner (Shibata et al., 2012; Dillon et al., 2015; Paulsen et al., 2018, 2019, 2021).

Despite intensive studies of DSBs in neuroscience, to my knowledge, no one has yet proposed or described activity-induced eccDNAs. Circular DNAs are expected to be especially stable (e.g., are resistant to nucleases such as DNase I), so eccDNAs may potentially help regulate long-term persistent changes in the phenotype of neurons—their firing behavior and expression of proteins and RNAs. If stable, the amount of extrachromosomal DNA produced over time would have a direct relationship to the long-term firing behavior of the neuron, providing a type of cellular “ticketing” or “genomic indexing” (Griffith and Mahler, 1969; Newman et al., 2017; Ueberham and Arendt, 2020; Giuditta et al., 2023).

## How might activity-induced eccDNAs mediate long-term responses to neuronal activation?

1. There is evidence from non-neural systems that extrachromosomal circular DNAs can serve as templates for the transcription of non-coding RNAs, including small inhibitory siRNAs and RNAs that interact with microRNAs (Allen et al., 2017; Paulsen et al., 2019), and if so, they could regulate gene expression within the neuron. Insofar as these eccDNAs arise from the activity-induced DSB sites, they would be expected to contain unique gene sequences related to activity-induced and synaptic genes and hence to regulate aspects of learning, memory and synaptic plasticity.
2. Another consequence of neuronal activation is the secretion of small vesicles called “exosomes” which contain specific proteins and RNAs, and which transfer signals to neighboring neurons (either presynaptic or postsynaptic partners) or nearby astrocytes, oligodendroglial cells or microglia (Smalheiser, 2007; Chivet et al., 2013; Gurung et al., 2021). It is known that exosomes can interact with recipient cells by binding surface receptors and by being incorporated into the cytoplasm and nucleus of the recipient cells (Gurung et al., 2021). Exosomal proteins and RNAs, including microRNAs, can regulate gene expression within the recipient cells. Extracellular DNA is associated with exosomes, but much of this appears to be contamination from dying or damaged cells, insofar as it is linear, covers the entire genome, and predominantly coats the outside of the exosomal membranes. Nevertheless, there is evidence that DNA is also contained within the exosomes and can be functionally transferred to recipient cells [(Peters and Pretorius, 2011; Waldenström et al., 2012; Cai et al., 2013, 2016; Fischer et al., 2016; Ono et al., 2019; Lichá et al., 2023), see also (Thakur et al., 2014; Lázaro-Ibáñez et al., 2019)]. If indeed activity-induced eccDNAs are formed within neurons, they may also be packaged within the exosomes, and transferred within recipient cells. Thus mobile DNAs may not simply have roles within the cell that produces it, but provide signals that regulate neighboring cells as well.
3. Our proposal may also relate to a third independent line of research: there is evidence that multiple copies of LINE-1 repeat sequences are inserted within hippocampal neurons, enriched in transcribed neuronal stem cell enhancers and hippocampus genes (Upton et al., 2015). The insertions may not only occur within neural precursor cells (Muotri et al., 2005), rather, various types of DNA sequences can be inserted into the chromosomes of postmitotic (non-dividing) neurons, including LINE-1 repeat elements (Macia et al., 2017; Newman et al., 2017) and retrotranscribed cellular RNAs (Giuditta and Casalino, 2020; Kaeser and Chun, 2020; Ueberham and Arendt, 2020; Giuditta et al., 2023), and at least some DNA insertions may occur at DSB sites (Ono et al., 2015; Newman et al., 2017). Exosome-associated DNA (treated with DNase I to remove linear adsorbent contaminants) has also been shown to incorporate into the chromosomes of non-neural recipient cells [(Cai et al., 2013, 2016; Fischer et al., 2016; Ono et al., 2019), see also (Emamalipour et al., 2020)]. DNA insertions alter genomic sequences in individual cells, and have a variety of possible consequences—changing the transcription, splicing and even coding of genes (Alt and Schwer, 2018). In this context, if activity-induced eccDNAs are produced at one time point, they would be a natural type of marker sequence to be inserted into distal regions of chromosomes of the same neuron, following a later activation that produces new DSBs. Thus, as the neuron is activated repeatedly, not only would the amount of activity-induced eccDNAs accumulate in the cell, but the number of insertions into chromosomes may accumulate as well. DNA insertions into genes in general, and immediate-early genes in particular, may tend to inactivate their expression—but in our scenario this should be helpful, not detrimental, to neuronal health since it could serve as a form of homeostasis against over-excitation (Newman et al., 2017).

## How to test the hypothesis

The hypothesis can, in principle, be tested using existing methods of DNA isolation and sequencing (Chiu et al., 2020; Kumar et al., 2020; Zhang et al., 2021; Mann et al., 2022; Jiang et al., 2023; Wang et al., 2023) and standard methods of mouse cortical neuron culture *in vitro* (Mukherjee et al., 2023) and fear conditioning paradigms *in vivo* (Pinna and Rasmusson, 2014; Locci and Pinna, 2019). The experiments comprise six steps:

1. Optimize or adapt methods for isolation and sequencing of extrachromosomal DNAs sufficient to detect eccDNAs within cultured mouse cortical neurons and intact mouse hippocampus. Beyond sequencing the entire pool of eccDNAs, it would be desirable to have a way to selectively tag only those eccDNAs that are formed during neuronal activation, to distinguish them from other eccDNAs (e.g., those expressed constitutively or deriving from damaged or stressed cells). DSB repair involves some incorporation of nucleotides into the repair site and presumably into the eccDNAs as well, at least at junctional regions. Thus, incubating cultured neurons during activation with biotin-dUTP, to replace normal dTTP, should incorporate biotin-dU analogs selectively into activity-induced eccDNAs and chromosomal DSB repair sites. The use of biotin-dUTP should allow subsequent biotin pull-down of the dU-containing eccDNAs using streptavidin beads (e.g., Lei et al., 2023).
2. Test whether acute activation of cultured mouse cortical neurons (via depolarization by treatment with NMDA, high K+ or bicuculline) causes rapid, specific formation of extrachromosomal circular DNAs (eccDNAs) arising at sites of double-strand breaks (DSBs), particularly those associated with immediate-early genes. Are eccDNAs are elicited with a time-course that follows DSB formation and repair? Is eccDNA formation dependent upon DSB formation and repair, as evidenced by inhibition when DSB formation is suppressed by agents (e.g., ICRF-193 or ICRF-187) that inhibit topoisomerase IIb? Do their sequences derive from activity-induced genes? Can they be labeled selectively using biotin-dUTP during activation?
3. Test whether eccDNAs will be acutely generated in the hippocampus of mice trained in contextual fear conditioning, or in mice exploring a novel environment. Will they be dependent upon DSB formation and repair? Will they persist for days or even weeks after their formation? Will their levels correlate with aspects of long-term memory such as extinction and reconsolidation?
4. Test whether the appearance of activity-induced eccDNAs is accompanied by the appearance of specific regulatory RNAs, whose sequences correspond to (and are putatively transcribed from) the eccDNAs. These may include:
  • small RNAs in the length ranges of 15–45 bases, encompassing microRNAs, siRNAs and other ncRNAs such as piRNAs (e.g., Smalheiser et al., 2011; Lugli et al., 2012, 2015; Smalheiser, 2012),
  • larger RNAs (including mRNAs and lncRNAs),
  • circular RNAs. Circular RNAs are particularly intriguing since some have well documented roles as microRNA “sponges” (i.e., binding and hence inactivating specific free microRNAs at multiple sites) and there is evidence that they can be transcribed from circular DNAs (Allen et al., 2017; Paulsen et al., 2019).
  If so, do these RNAs regulate specific target mRNAs and proteins? Do these RNAs and their targets regulate aspects of synaptic plasticity, learning and memory?
5. Test whether exosomes released from stimulated cultured mouse cortical neurons contain activity-induced eccDNAs. If so, can they be transferred to recipient cells? For example, can mouse neurons cultured on one side of a microporous membrane (George et al., 2017) transfer mouse eccDNAs to human-induced pluripotent derived neuronal cells cultured on the other side? Can one define a pathway for transport of eccDNAs from the nucleus to cytoplasmic sites wherein exosomes are loaded?
6. Test whether activity-induced eccDNAs are incorporated into distant sites in the chromosomes of a neuron, especially at DSBs formed during subsequent activation events. This is the most difficult step to assess, but may be approached using the biotin-dUTP technique to label activity-induced eccDNAs during a short interval following activation, i.e., stimulating cultured neurons with NMDA while incorporating biotin-dU into eccDNAs selectively within the subsequent 2 h. After a further interval of 24 h, to allow recovery of the cells and washout of the biotin-dUTP, the cultures can be stimulated a second time to induce new DSB sites to form, and 2 h later, the neurons’ chromosomal DNA can be isolated, fragmented by sonication or using rare-cutting restriction enzymes to produce fragments of a more controlled average size suitable for sequencing, and the fragments exposed to biotin pulldown to selectively isolate chromosomal DNA fragments that contain biotin-dU, followed by sequencing (Smits and Faulkner, 2023).
  The biotin-containing fragments should comprise a mix of DSB sites formed and repaired during the first activation stimulus, and fragments containing eccDNAs that were inserted into chromosomes during the second activation stimulus. The two can be distinguished by the distance between biotin-containing inverse repeats—very close for DSB sites, vs. hundreds to thousands of bases for eccDNA inserts. As well, sequencing of the fragments should directly show eccDNA inserts embedded within distal chromosomal regions, far from the original site of origin of the eccDNA sequences.

## Conclusion

In summary, I hypothesize the existence of a new, fundamental (and somewhat heretical) biochemical pathway of mobile circular DNAs regulating the long-term behavior of neurons in the brain. This hypothesis elegantly ties together several hitherto separate and somewhat puzzling findings within neuroscience, including the observations that:

• stimulating neuronal activity causes neurons to make double-stranded breaks at specific sites within their genomic DNA;
• stimulating activity causes an increase in transposition of sequences (repeat sequences and retrotranscribed RNAs) that insert into genomic DNA even in postmitotic non-dividing neurons;
• stimulating neuronal activity causes an increase in secretion of small vesicles (“exosomes”) that in addition to containing specific proteins and RNAs, also are associated with DNA.

Such a discovery would offer a paradigm shift comparable to the discoveries of gene splicing, microRNAs, or transposable elements. Activity-induced eccDNAs may provide new experimental tools for investigating and perturbing neuronal physiology, and may provide biomarkers reflecting the state of neuronal activity and health.

Furthermore, neurons also exhibit an increase in DSBs during aging and in a variety of neurodegenerative diseases (e.g., Zhu et al., 2019; Konopka and Atkin, 2022), though these DSBs may not all be created nor repaired in the same manner as the activity-induced DSBs. In the future, it will be important to compare eccDNAs formed under physiological vs. pathological conditions, and to learn whether the physiological pathway proposed here contributes to the pathophysiology of Alzheimer’s disease and other neurodegenerative diseases (Konopka and Atkin, 2022). If so, targeting the turnover of eccDNAs or their RNA transcripts may provide a new therapeutic strategy.

## Data availability statement

The original contributions presented in this study are included in this article/supplementary material, further inquiries can be directed to the corresponding author.

## Author contributions

NS: Writing – original draft.

## References

[B1] Allen S. E. Hug I. Pabian S. Rzeszutek I. Hoehener C. Nowacki M. (2017). Circular concatemers of ultra-short DNA segments produce regulatory RNAs. *Cell* 168 990–999.e7. 10.1016/j.cell.2017.02.020 28283070 PMC5346157

[B2] Alt F. W. Schwer B. (2018). DNA double-strand breaks as drivers of neural genomic change, function, and disease. *DNA Repair.* 71 158–163. 10.1016/j.dnarep.2018.08.019 30195640 PMC6340756

[B3] Bellesi M. Bushey D. Chini M. Tononi G. Cirelli C. (2016). Contribution of sleep to the repair of neuronal DNA double-strand breaks: Evidence from flies and mice. *Sci. Rep.* 6:36804. 10.1038/srep36804 27830758 PMC5103291

[B4] Cai J. Han Y. Ren H. Chen C. He D. Zhou L. (2013). Extracellular vesicle-mediated transfer of donor genomic DNA to recipient cells is a novel mechanism for genetic influence between cells. *J. Mol. Cell Biol.* 5 227–238. 10.1093/jmcb/mjt011 23580760 PMC3733418

[B5] Cai J. Wu G. Jose P. A. Zeng C. (2016). Functional transferred DNA within extracellular vesicles. *Exp. Cell Res.* 349 179–183. 10.1016/j.yexcr.2016.10.012 27751837

[B6] Calderwood S. K. (2016). A critical role for topoisomerase IIb and DNA double strand breaks in transcription. *Transcription* 7 75–83. 10.1080/21541264.2016.1181142 27100743 PMC4984685

[B7] Chiu R. W. Dutta A. Hensson A. G. Lo Y. M. Mischel P. Regenberg B. (2020). What Is Extrachromosomal Circular DNA and What Does It Do? *Clin Chem.* 66 754–759. 10.1093/clinchem/hvaa096 32470125 PMC7259478

[B8] Chivet M. Javalet C. Hemming F. Pernet-Gallay K. Laulagnier K. Fraboulet S. (2013). Exosomes as a novel way of interneuronal communication. *Biochem. Soc. Trans.* 41 241–244. 10.1042/BST20120266 23356290

[B9] Crowe S. L. Movsesyan V. A. Jorgensen T. J. Kondratyev A. (2006). Rapid phosphorylation of histone H2A.X following ionotropic glutamate receptor activation. *Eur. J. Neurosci.* 23 2351–2361. 16706843 10.1111/j.1460-9568.2006.04768.x PMC1534119

[B10] Delint-Ramirez I. Konada L. Heady L. Rueda R. Jacome A. S. Marlin E. (2022). Calcineurin dephosphorylates topoisomerase IIβ and regulates the formation of neuronal-activity-induced DNA breaks. *Mol. Cell.* 82 3794–3809.e8. 10.1016/j.molcel.2022.09.012 36206766 PMC9990814

[B11] Dillon L. W. Kumar P. Shibata Y. Wang Y. H. Willcox S. Griffith J. D. (2015). Production of extrachromosomal MicroDNAs is linked to mismatch repair pathways and transcriptional activity. *Cell Rep.* 11 1749–1759. 10.1016/j.celrep.2015.05.020 26051933 PMC4481157

[B12] Emamalipour M. Seidi K. Zununi Vahed S. Jahanban-Esfahlan A. Jaymand M. Majdi H. (2020). Horizontal gene transfer: From evolutionary flexibility to disease progression. *Front. Cell Dev. Biol.* 8:229. 10.3389/fcell.2020.00229 32509768 PMC7248198

[B13] Fischer S. Cornils K. Speiseder T. Badbaran A. Reimer R. Indenbirken D. (2016). Indication of horizontal DNA gene transfer by extracellular vesicles. *PLoS One* 11:e0163665. 10.1371/journal.pone.0163665 27684368 PMC5042424

[B14] George J. H. Waller S. T. Ye H. (2017). “4.13 Use and manipulation of microporous membranes in mammalian cell cultures,” in *Comprehensive membrane science and engineering*, Second Edn, (Amsterdam: Elsevier), 272–292.

[B15] Giuditta A. Casalino J. (2020). Sequences of reverse transcribed brain DNA are modified by learning. *Front. Mol. Neurosci.* 13:57. 10.3389/fnmol.2020.00057 32410960 PMC7199793

[B16] Giuditta A. Zucconi G. G. Sadile A. (2023). Brain metabolic DNA: A long story and some conclusions. *Mol. Neurobiol.* 60 228–234. 10.1007/s12035-022-03030-y 36251232

[B17] Griffith J. S. Mahler H. R. (1969). DNA ticketing theory of memory. *Nature* 223 580–582. 10.1038/223580a0 5799529

[B18] Guo J. Zhang Z. Li Q. Chang X. Liu X. (2023). TeCD: The eccDNA Collection Database for extrachromosomal circular DNA. *BMC Genomics.* 24:47. 10.1186/s12864-023-09135-5 36707765 PMC9881285

[B19] Gurung S. Perocheau D. Touramanidou L. Baruteau J. (2021). The exosome journey: From biogenesis to uptake and intracellular signalling. *Cell Commun. Signal.* 19:47. 10.1186/s12964-021-00730-1 33892745 PMC8063428

[B20] Jiang R. Yang M. Zhang S. Huang M. (2023). Advances in sequencing-based studies of microDNA and ecDNA: Databases, identification methods, and integration with single-cell analysis. *Comput. Struct. Biotechnol. J.* 21 3073–3080. 10.1016/j.csbj.2023.05.017 37273851 PMC10238454

[B21] Kaeser G. Chun J. (2020). Brain cell somatic gene recombination and its phylogenetic foundations. *J. Biol. Chem.* 295 12786–12795. 10.1074/jbc.REV120.009192 32699111 PMC7476723

[B22] Konopka A. Atkin J. D. (2022). The role of DNA damage in neural plasticity in physiology and neurodegeneration. *Front. Cell Neurosci.* 16:836885. 10.3389/fncel.2022.836885 35813507 PMC9259845

[B23] Kumar P. Kiran S. Saha S. Su Z. Paulsen T. Chatrath A. (2020). ATAC-seq identifies thousands of extrachromosomal circular DNA in cancer and cell lines. *Sci. Adv.* 6:eaba2489. 10.1126/sciadv.aba2489 32440553 PMC7228742

[B24] Lázaro-Ibáñez E. Lässer C. Shelke G. V. Crescitelli R. Jang S. C. Cvjetkovic A. (2019). DNA analysis of low- and high-density fractions defines heterogeneous subpopulations of small extracellular vesicles based on their DNA cargo and topology. *J. Extracell Vesicles* 8:1656993. 10.1080/20013078.2019.1656993 31497265 PMC6719264

[B25] Lei Z. Meng H. Rao X. Zhao H. Yi C. (2023). Detect-seq, a chemical labeling and biotin pull-down approach for the unbiased and genome-wide off-target evaluation of programmable cytosine base editors. *Nat. Protoc.* 18 2221–2255. 10.1038/s41596-023-00837-4 37277562

[B26] Li X. Marshall P. R. Leighton L. J. Zajaczkowski E. L. Wang Z. Madugalle S. U. (2019). The DNA repair-associated protein Gadd45γ regulates the temporal coding of immediate early gene expression within the prelimbic prefrontal cortex and is required for the consolidation of associative fear memory. *J. Neurosci.* 39 970–983. 10.1523/JNEUROSCI.2024-18.2018 30545945 PMC6363930

[B27] Lichá K. Pastorek M. Repiská G. Celec P. Konečná B. (2023). Investigation of the presence of DNA in human blood plasma small extracellular vesicles. *Int. J. Mol. Sci.* 24:5915. 10.3390/ijms24065915 36982989 PMC10051167

[B28] Ling X. Han Y. Meng J. Zhong B. Chen J. Zhang H. (2021). Small extrachromosomal circular DNA (eccDNA): Major functions in evolution and cancer. *Mol. Cancer* 20:113. 10.1186/s12943-021-01413-8 34479546 PMC8414719

[B29] Locci A. Pinna G. (2019). Stimulation of peroxisome proliferator-activated receptor-α by N-palmitoylethanolamine engages allopregnanolone biosynthesis to modulate emotional behavior. *Biol. Psychiatry* 85 1036–1045. 10.1016/j.biopsych.2019.02.006 30955840

[B30] Lugli G. Cohen A. M. Bennett D. A. Shah R. C. Fields C. J. Hernandez A. G. (2015). Plasma exosomal miRNAs in persons with and without Alzheimer disease: Altered expression and prospects for biomarkers. *PLoS One.* 10:e0139233. 10.1371/journal.pone.0139233 26426747 PMC4591334

[B31] Lugli G. Larson J. Demars M. P. Smalheiser N. R. (2012). Primary microRNA precursor transcripts are localized at post-synaptic densities in adult mouse forebrain. *J. Neurochem.* 123 459–466. 10.1111/j.1471-4159.2012.07921.x 22897173

[B32] Macia A. Widmann T. J. Heras S. Ayllon V. Sanchez L. Benkaddour-Boumzaouad M. (2017). Engineered LINE-1 retrotransposition in nondividing human neurons. *Genome Res.* 27 335–348. 10.1101/gr.206805.116 27965292 PMC5340962

[B33] Madabhushi R. Gao F. Pfenning A. R. Pan L. Yamakawa S. Seo J. (2015). Activity-induced DNA breaks govern the expression of neuronal early-response genes. *Cell* 161 1592–1605. 10.1016/j.cell.2015.05.032 26052046 PMC4886855

[B34] Mann L. Seibt K. M. Weber B. Heitkam T. (2022). ECCsplorer: A pipeline to detect extrachromosomal circular DNA (eccDNA) from next-generation sequencing data. *BMC Bioinform.* 23:40. 10.1186/s12859-021-04545-2 35030991 PMC8760651

[B35] Mukherjee S. Malik P. Mukherjee T. K. (2023). “Culture of neuron and glia cells,” in *Practical approach to mammalian cell and organ culture*, eds Mukherjee T. K. Malik P. Mukherjee S. (Singapore: Springer), 10.1007/978-981-19-1731-8_10-2

[B36] Muotri A. R. Chu V. T. Marchetto M. C. Deng W. Moran J. V. Gage F. H. (2005). Somatic mosaicism in neuronal precursor cells mediated by L1 retrotransposition. *Nature* 435 903–910. 10.1038/nature03663 15959507

[B37] Navabpour S. Rogers J. McFadden T. Jarome T. J. (2020). DNA double-strand breaks are a critical regulator of fear memory reconsolidation. *Int. J. Mol. Sci.* 21:8995. 10.3390/ijms21238995 33256213 PMC7730899

[B38] Newman A. G. Bessa P. Tarabykin V. Singh P. B. (2017). Activity-DEPendent transposition. *EMBO Rep.* 18 346–348. 10.15252/embr.201643797 28219904 PMC5331197

[B39] Ono R. Ishii M. Fujihara Y. Kitazawa M. Usami T. Kaneko-Ishino T. (2015). Double strand break repair by capture of retrotransposon sequences and reverse-transcribed spliced mRNA sequences in mouse zygotes. *Sci. Rep.* 5:12281. 10.1038/srep12281 26216318 PMC4516963

[B40] Ono R. Yasuhiko Y. Aisaki K. I. Kitajima S. Kanno J. Hirabayashi Y. (2019). Exosome-mediated horizontal gene transfer occurs in double-strand break repair during genome editing. *Commun. Biol.* 2:57. 10.1038/s42003-019-0300-2 30775458 PMC6368560

[B41] Paulsen T. Kumar P. Koseoglu M. M. Dutta A. (2018). Discoveries of extrachromosomal circles of DNA in normal and tumor cells. *Trends Genet.* 34 270–278. 10.1016/j.tig.2017.12.010 29329720 PMC5881399

[B42] Paulsen T. Malapati P. Shibata Y. Wilson B. Eki R. Benamar M. (2021). MicroDNA levels are dependent on MMEJ, repressed by c-NHEJ pathway, and stimulated by DNA damage. *Nucleic Acids Res.* 49 11787–11799. 10.1093/nar/gkab984 34718766 PMC8599734

[B43] Paulsen T. Shibata Y. Kumar P. Dillon L. Dutta A. (2019). Small extrachromosomal circular DNAs, microDNA, produce short regulatory RNAs that suppress gene expression independent of canonical promoters. *Nucleic Acids Res.* 47 4586–4596. 10.1093/nar/gkz155 30828735 PMC6511871

[B44] Peters D. L. Pretorius P. J. (2011). Origin, translocation and destination of extracellular occurring DNA–a new paradigm in genetic behaviour. *Clin. Chim Acta.* 412 806–811. 10.1016/j.cca.2011.01.026 21277292

[B45] Pinna G. Rasmusson A. M. (2014). Ganaxolone improves behavioral deficits in a mouse model of post-traumatic stress disorder. *Front. Cell Neurosci.* 8:256. 10.3389/fncel.2014.00256 25309317 PMC4161165

[B46] Pollina E. A. Gilliam D. T. Landau A. T. Lin C. Pajarillo N. Davis C. P. (2023). A NPAS4-NuA4 complex couples synaptic activity to DNA repair. *Nature* 614 732–741. 10.1038/s41586-023-05711-7 36792830 PMC9946837

[B47] Shibata Y. Kumar P. Layer R. Willcox S. Gagan J. R. Griffith J. D. (2012). Extrachromosomal microDNAs and chromosomal microdeletions in normal tissues. *Science* 336 82–86. 22403181 10.1126/science.1213307 PMC3703515

[B48] Smalheiser N. R. (2007). Exosomal transfer of proteins and RNAs at synapses in the nervous system. *Biol. Direct.* 2:35. 10.1186/1745-6150-2-35 18053135 PMC2219957

[B49] Smalheiser N. R. (2012). The search for endogenous siRNAs in the mammalian brain. *Exp Neurol.* 235 455–463. 10.1016/j.expneurol.2011.10.015 22062046 PMC3291744

[B50] Smalheiser N. R. Lugli G. Thimmapuram J. Cook E. H. Larson J. (2011). Endogenous siRNAs and noncoding RNA-derived small RNAs are expressed in adult mouse hippocampus and are up-regulated in olfactory discrimination training. *RNA.* 17 166–181. 10.1261/rna.2123811 21045079 PMC3004058

[B51] Smits N. Faulkner G. J. (2023). Nanopore sequencing to identify transposable element insertions and their epigenetic modifications. *Methods Mol. Biol.* 2607 151–171. 10.1007/978-1-0716-2883-6_9 36449163

[B52] Stott R. T. Kritsky O. Tsai L. H. (2021). Profiling DNA break sites and transcriptional changes in response to contextual fear learning. *PLoS One* 16:e0249691. 10.1371/journal.pone.0249691 34197463 PMC8248687

[B53] Suberbielle E. Sanchez P. E. Kravitz A. V. Wang X. Ho K. Eilertson K. (2013). Physiologic brain activity causes DNA double-strand breaks in neurons, with exacerbation by amyloid-β. *Nat. Neurosci.* 16 613–621. 10.1038/nn.3356 23525040 PMC3637871

[B54] Thakur B. K. Zhang H. Becker A. Matei I. Huang Y. Costa-Silva B. (2014). Double-stranded DNA in exosomes: A novel biomarker in cancer detection. *Cell Res.* 24 766–769. 10.1038/cr.2014.44 24710597 PMC4042169

[B55] Ueberham U. Arendt T. (2020). Genomic indexing by somatic gene recombination of mRNA/ncRNA - does it play a role in genomic mosaicism, memory formation, and Alzheimer’s disease? *Front. Genet.* 11:370. 10.3389/fgene.2020.00370 32411177 PMC7200996

[B56] Upton K. R. Gerhardt D. J. Jesuadian J. S. Richardson S. R. Sánchez-Luque F. J. Bodea G. O. (2015). Ubiquitous L1 mosaicism in hippocampal neurons. *Cell* 161 228–239. 10.1016/j.cell.2015.03.026 25860606 PMC4398972

[B57] Waldenström A. Gennebäck N. Hellman U. Ronquist G. (2012). Cardiomyocyte microvesicles contain DNA/RNA and convey biological messages to target cells. *PLoS One* 7:e34653. 10.1371/journal.pone.0034653 22506041 PMC3323564

[B58] Wang Y. Wang M. Zhang Y. (2023). Purification, full-length sequencing and genomic origin mapping of eccDNA. *Nat. Protoc.* 18 683–699. 10.1038/s41596-022-00783-7 36517607

[B59] Weber Boutros S. Unni V. K. Raber J. (2022). An adaptive role for DNA double-strand breaks in hippocampus-dependent learning and memory. *Int. J. Mol. Sci.* 23:8352. 10.3390/ijms23158352 35955487 PMC9368779

[B60] Zada D. Bronshtein I. Lerer-Goldshtein T. Garini Y. Appelbaum L. (2019). Sleep increases chromosome dynamics to enable reduction of accumulating DNA damage in single neurons. *Nat. Commun.* 10:895. 10.1038/s41467-019-08806-w 30837464 PMC6401120

[B61] Zada D. Sela Y. Matosevich N. Monsonego A. Lerer-Goldshtein T. Nir Y. (2021). Parp1 promotes sleep, which enhances DNA repair in neurons. *Mol. Cell.* 81 4979–4993.e7. 10.1016/j.molcel.2021.10.026 34798058 PMC8688325

[B62] Zhang P. Peng H. Llauro C. Bucher E. Mirouze M. (2021). ecc_finder: A robust and accurate tool for detecting extrachromosomal circular DNA from sequencing data. *Front. Plant Sci.* 12:743742. 10.3389/fpls.2021.743742 34925397 PMC8672306

[B63] Zhao Y. Yu L. Zhang S. Su X. Zhou X. (2022). Extrachromosomal circular DNA: Current status and future prospects. *Elife* 11:e81412. 10.7554/eLife.81412 36256570 PMC9578701

[B64] Zhong T. Wang W. Liu H. Zeng M. Zhao X. Guo Z. (2023). eccDNA Atlas: A comprehensive resource of eccDNA catalog. *Brief Bioinform.* 24:bbad037. 10.1093/bib/bbad037 36757087

[B65] Zhu L. S. Wang D. Q. Cui K. Liu D. Zhu L. Q. (2019). Emerging perspectives on DNA double-strand breaks in neurodegenerative diseases. *Curr. Neuropharmacol.* 17 1146–1157. 10.2174/1570159X17666190726115623 31362659 PMC7057204

